# Blockade of L-type Ca^2+^ channel attenuates doxorubicin-induced cardiomyopathy via suppression of CaMKII-NF-κB pathway

**DOI:** 10.1038/s41598-019-46367-6

**Published:** 2019-07-08

**Authors:** Soichiro Ikeda, Shouji Matsushima, Kosuke Okabe, Masataka Ikeda, Akihito Ishikita, Tomonori Tadokoro, Nobuyuki Enzan, Taishi Yamamoto, Masashi Sada, Hiroko Deguchi, Sachio Morimoto, Tomomi Ide, Hiroyuki Tsutsui

**Affiliations:** 1 Department of Cardiovascular Medicine, Faculty of Medical Sciences, Kyushu University, Fukuoka, Japan; 2 Department of Cardiovascular Medicine, Kyushu University Hospital, Fukuoka, Japan; 3 Department of Health Sciences Fukuoka, International University of Health and Welfare, Okawa, Japan; 4 Department of Experimental and Clinical Cardiovascular Medicine, Graduate School of Medical Sciences, Kyushu University, Fukuoka, Japan

**Keywords:** Cardiomyopathies, Heart failure

## Abstract

Ca^2+^/calmodulin-dependent protein kinase II (CaMKII) and nuclear factor-kappa B (NF-κB) play crucial roles in pathogenesis of doxorubicin (DOX)-induced cardiomyopathy. Their activities are regulated by intracellular Ca^2+^. We hypothesized that blockade of L-type Ca^2+^ channel (LTCC) could attenuate DOX-induced cardiomyopathy by regulating CaMKII and NF-κB. DOX activated CaMKII and NF-κB through their phosphorylation and increased cleaved caspase 3 in cardiomyocytes. Pharmacological blockade or gene knockdown of LTCC by nifedipine or small interfering RNA, respectively, suppressed DOX-induced phosphorylation of CaMKII and NF-κB and apoptosis in cardiomyocytes, accompanied by decreasing intracellular Ca^2+^ concentration. Autocamtide 2-related inhibitory peptide (AIP), a selective CaMKII inhibitor, inhibited DOX-induced phosphorylation of NF-κB and cardiomyocyte apoptosis. Inhibition of NF-κB activity by ammonium pyrrolidinedithiocarbamate (PDTC) suppressed DOX-induced cardiomyocyte apoptosis. DOX-treatment (18 mg/kg via intravenous 3 injections over 1 week) increased phosphorylation of CaMKII and NF-κB in mouse hearts. Nifedipine (10 mg/kg/day) significantly suppressed DOX-induced phosphorylation of CaMKII and NF-κB and cardiomyocyte injury and apoptosis in mouse hearts. Moreover, it attenuated DOX-induced left ventricular dysfunction and dilatation. Our findings suggest that blockade of LTCC attenuates DOX-induced cardiomyocyte apoptosis via suppressing intracellular Ca^2+^ elevation and activation of CaMKII-NF-κB pathway. LTCC blockers might be potential therapeutic agents against DOX-induced cardiomyopathy.

## Introduction

Doxorubicin (DOX) is an anthracycline antibiotic, which is widely used and efficacious chemotherapeutic drug for hematological malignancies and solid tumors. However, it has dose-dependent cardiotoxicity such as loss of cardiomyocytes and cardiac dysfunction, thereby limiting its clinical use^1^. DOX-induced cardiomyopathy has poor prognosis compared with dilated cardiomyopathy or ischemic cardiomyopathy^2^. Therefore, the establishment of effective therapeutic strategy for DOX-induced cardiomyopathy is urgently needed.

Cardiomyocyte death such as apoptosis is a critical process implicated in DOX-induced cardiomyopathy^3,4^. A number of studies have suggested the molecular mechanisms of this disease state, including alteration of transcription by topoisomerase II^5^, mitochondrial iron accumulation^6^, oxidative stress such as lipid peroxidation^7^ and protein nitrosylation^8^, mitochondrial dysfunction^9^, and Ca^2+^ handling abnormality^10,11^. However, the fundamental mechanism underlying DOX-induced cardiomyocyte apoptosis remains to be fully elucidated.

Ca^2+^/calmodulin-dependent protein kinase II (CaMKII) is a multifunctional serine/threonine-specific protein kinase, which is intimately involved in signaling cascades of cardiomyocyte survival and death^12^. A recent evidence suggests that activation of CaMKII critically mediates DOX-induced cardiomyocyte death and cardiac dysfunction^13^. As its name implies, CaMKII activity is dependent of intracellular Ca^2+^ ^14^. Among potential sources of intracellular Ca^2+^, L-type Ca^2+^ channel (LTCC) is a major contributor to intracellular Ca^2+^ in cardiomyocytes^15,16^. To date, amlodipine, a LTCC blocker, has been reported to attenuate DOX-induced cardiomyocyte apoptosis *in vitro* by unknown mechanism^17^. In addition, nifedipine, another LTCC blocker, is known to inhibit pathological cardiac hypertrophy by suppressing CaMKII activity in cardiomyocytes^18^. These findings raise a possibility that blockade of LTCC exerts a protective role against DOX-induced cardiomyopathy by suppressing CaMKII-mediated cardiomyocyte apoptosis.

Nuclear factor-kappa B (NF-κB) is a transcriptional factor regulating genes associated with stress responses including apoptosis^19^. Several studies have demonstrated that NF-κB is a critical mediator of DOX-induced cardiotoxicity^20,21^. NF-κB is composed of multiple subunits such as RelA (p65), RelB, c-Rel, NF-kB1 (p50) and NF-κB2 (p52) and phosphorylation of p65 promotes NF-κB activity^22,23^. Although, importantly, NF-kB activity is also known to be regulated by intracellular Ca^2+^ ^24^, no study has ever been performed to specifically examine the link between LTCC and NF-κB in cardiomyocytes.

A large number of animal experiments regarding DOX-induced cardiomyopathy have been performed by using a model of high-dose DOX treatment. This model is characterized by not only acute cardiotoxicity and cardiac dysfunction but also severe extra-cardiac phenotypes including malaise, anorexia, body weight loss, and non-cardiac death, resulting in conflicting interpretations with cardiac phenotype^25^ and leading to discrepant findings between animal experiment and clinical setting. Recently, a novel murine model of a low-dose DOX mimicking human DOX-induced cardiomyopathy has been reported, which causes modest but progressive cardiac dysfunction without severe non-cardiac effects^25,26^. This model is thought to be ideal for *in vivo* experiment regarding DOX-induced cardiomyopathy.

The present study aimed to determine whether blockade of LTCC attenuates DOX-induced cardiomyocyte apoptosis by suppressing intracellular Ca^2+^ levels and activities of CaMKII and NF-κB and whether it could ameliorate DOX-induced cardiomyopathy by using a low-dose DOX-treated model.

## Results

### DOX induced phosphorylation of CaMKII and NF-κB and increased cleaved caspase 3 in cardiomyocytes in vitro

**Figure 1:**
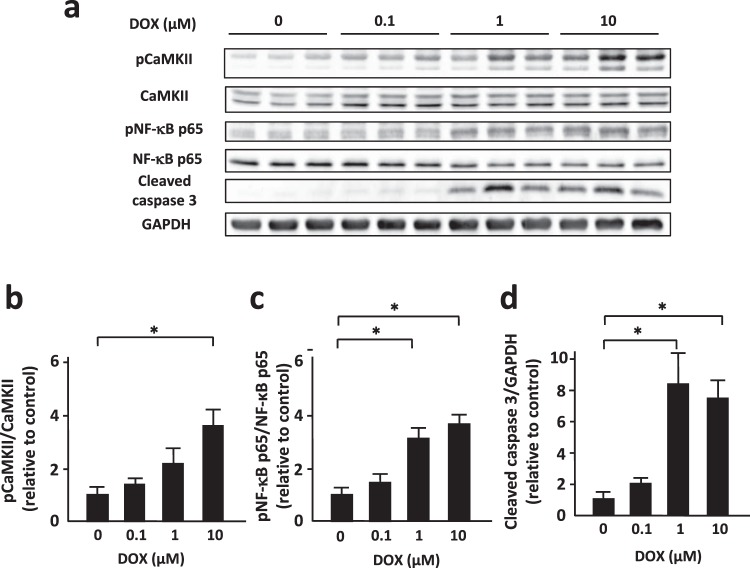
DOX induced phosphorylation of CaMKII and NF-κB and increased cleaved caspase 3 in cardiomyocytes in a dose-dependent manner. (**a**) Representative immunoblots of CaMKII, phosphorylated CaMKII, NF-κB, phosphorylated NF-κB, cleaved caspase 3, and GAPDH in cultured neonatal rat ventricular myocytes (NRVMs) treated with indicated concentrations of DOX. (**b**–**d**) Quantitative analysis of phosphorylated CaMKII, phosphorylated NF-κB, and cleaved caspase 3 in NRVMs treated with indicated concentration of DOX (n = 5). The experiment was conducted 2 times. *P < 0.05: post-hoc Tukey’s comparison test.

First, we examined the effect of doxorubicin (DOX) on regulation of CaMKII and NF-κB in neonatal rat ventricular myocytes (NRVMs). DOX increased phosphorylated CaMKII, an active form of CaMKII, in NRVMs in a dose-dependent manner with no significant changes in total CaMKII protein levels (Fig. 1a,b). Whereas DOX slightly, but not significantly, decreased NF-κB p65 protein levels, it increased phosphorylated NF-κB p65, an active form of NF-κB p65, in a dose-dependent manner (Fig. 1a,c). Consistent with phosphorylation of CaMKII and NF-κB p65, DOX increased cleaved caspase 3 protein levels in NRVMs (Fig. 1a,d). Moreover, DOX-induced phosphorylation of CaMKII and NF-κB p65 and increase in cleaved caspase 3 were time-dependent (Supplementary Fig. 1a–d). These data indicate that activation of CaMKII and NF-κB is intimately involved in DOX-induced apoptosis in cardiomyocytes.

### Blockade of LTCC suppressed DOX-induced phosphorylation of CaMKII and NF-κB and increases in cleaved caspase 3 in cardiomyocytes

**Figure 2:**
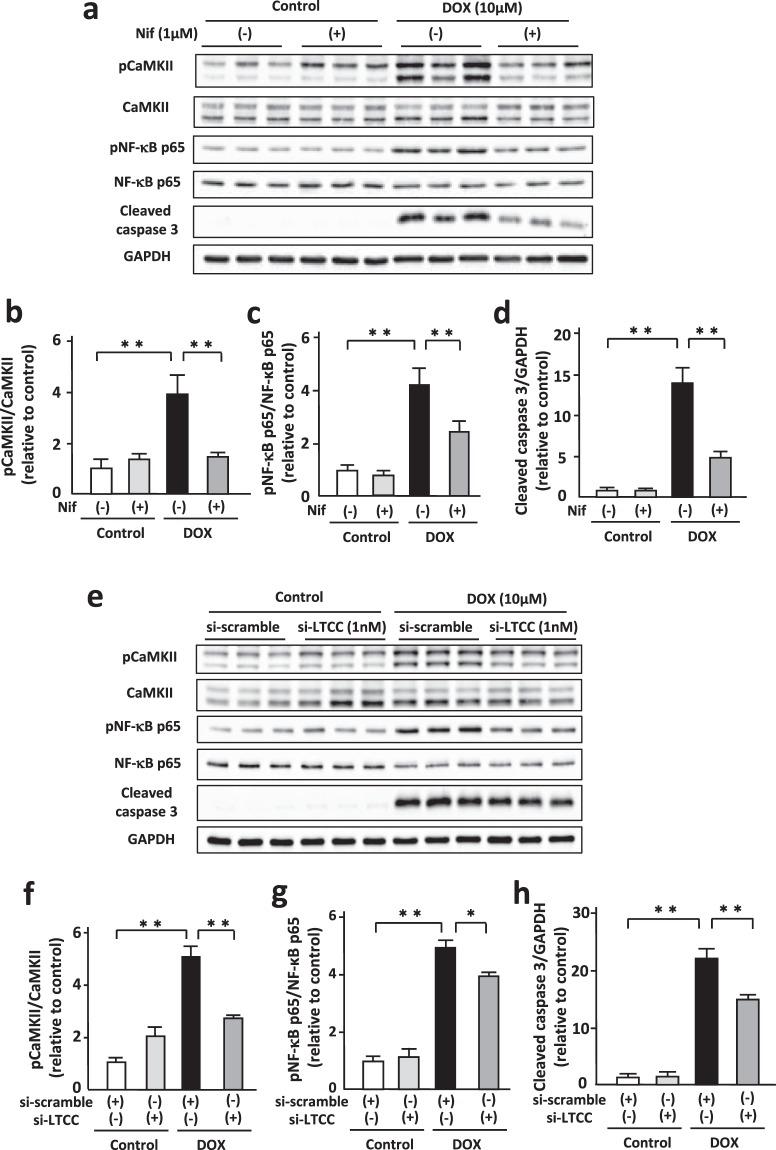
Blockade of LTCC suppressed DOX-induced phosphorylation of CaMKII and NF-κB and increases in cleaved caspase 3 in cardiomyocytes. (**a**) Representative immunoblots of CaMKII, phosphorylated CaMKII, NF-κB, phosphorylated NF-κB, cleaved caspase 3, and GAPDH in NRVMs treated with or without nifedipine (Nif, 1 μM) in the presence or absence of DOX (10 μM) for 24 hour (n = 5). The experiment was conducted 3 times. (**b**–**d**) Quantitative analysis of phosphorylated CaMKII, phosphorylated NF-κB, and cleaved caspase 3 in each group (n = 5). (**e**) Representative immunoblots of CaMKII, phosphorylated CaMKII, NF-κB, phosphorylated NF-κB, cleaved caspase 3, and GAPDH in NRVMs treated with indicated small interfering RNA (siRNA) in the presence or absence of DOX (10 μM) for 24 hour (n = 5). (**f**–**h**) Quantitative analysis of phosphorylated CaMKII, phosphorylated NF-κB, and cleaved caspase 3 in each group (n = 5). The experiment was conducted 3 times. *P < 0.05, **P < 0.01: post-hoc Tukey’s comparison test.

Next, we investigated the effect of L-type Ca^2+^ channel (LTCC) on DOX-induced activation of CaMKII and NF-κB. DOX increased phosphorylated LTCC, its active form, in NRVMs (Supplementary Fig. 2). Nifedipine (1 μM, 24 h) suppressed DOX-induced phosphorylation of CaMKII and NF-κB p65 in cardiomyocytes with no significant changes of their expression levels (Fig. 2a–c). Nifedipine attenuated DOX-induced increases in cleaved caspase 3 (Fig. 2a,d). Amlodipine (1 μM, 24 h), another LTCC channel blocker, also attenuated DOX-induced phosphorylation of CaMKII and NF-κB p65 and increases in cleaved caspase 3 (Supplementary Fig. 3a–d). To evaluate the direct effects of LTCC on CaMKII and NF-κB, we transduced small interfering RNA (siRNA) for LTCC into cardiomyocytes. Transduction of siRNA significantly decreased LTCC expression levels in cardiomyocytes (Supplementary Fig. 4). Downregulation of LTCC suppressed DOX-induced phosphorylation of CaMKII and NF-κB p65 (Fig. 2e–g). It also attenuated DOX-induced increases in cleaved caspase 3 (Fig. 2e,h). These data indicate that LTCC plays a crucial role in DOX-induced activation of CaMKII and NF-κB and apoptosis in cardiomyocytes.

### Blockade of LTCC attenuated DOX-induced cardiomyocyte apoptosis

**Figure 3:**
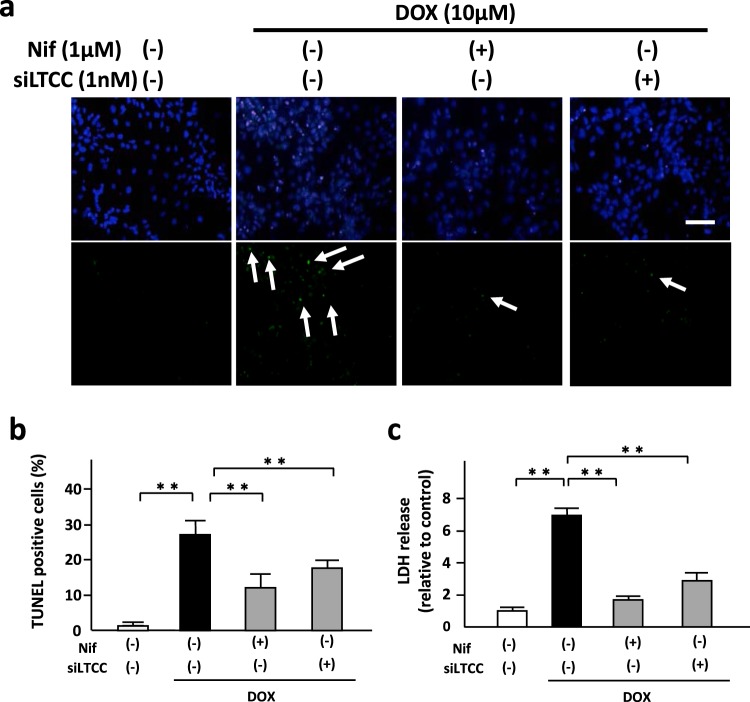
Blockade of LTCC attenuated DOX-induced cardiomyocyte apoptosis. (**a**,**b**) Apoptosis evaluated by TUNEL staining in NRVMs treated with nifedipine (Nif, 1 μM) or siRNA for LTCC (1 nM) in the presence or absence of DOX (10 μM) for 24 hours (n = 5–6). The experiment was conducted 3 times. Arrows indicate TUNEL-positive cells. (**c**) LDH release, a marker of cell death, evaluated by LDH cytotoxicity assay in NRVMs treated with nifedipine (Nif) or siRNA for LTCC in the presence or absence of DOX (10 μM) for 24 hours (n = 5–6). The experiment was conducted 3 times. **P < 0.01: post-hoc Tukey’s comparison test.

We investigated the role of LTCC on cardiomyocyte apoptosis evaluated by TUNEL staining. Both nifedipine (Fig. 3a,b) and amlodipine (Supplementary Fig. 3e) significantly attenuated DOX-induced cardiomyocyte apoptosis. They suppressed DOX-induced LDH release, a marker of cell death, from NRVMs (Fig. 3c and Supplementary Fig. 3f). In addition, downregulation of LTCC by siRNA also ameliorated DOX-induced cardiomyocyte apoptosis (Fig. 3a,b) and LDH releases in cardiomyocytes (Fig. 3c).

### Blockade of LTCC attenuated DOX-induced elevation of intracellular Ca^2+^ levels in cardiomyocytes

**Figure 4:**
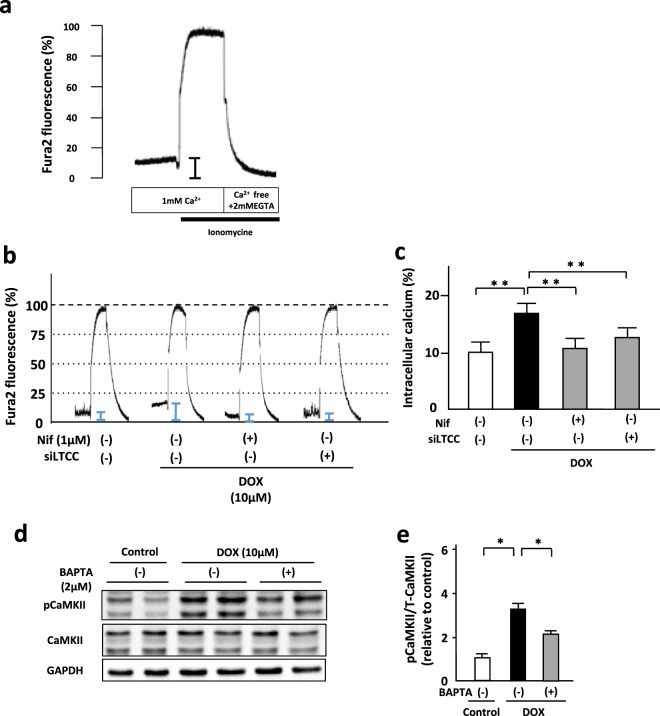
Blockade of LTCC attenuated DOX-induced elevation of intracellular Ca^2+^ levels in cardiomyocytes. (**a**) The resting levels of intracellular Ca^2+^ concentration in NRVMs were evaluated by fura-2 fluorometry. (**b**) A representative recording of fura-2 fluorometry in NRVMs treated with nifedipine (Nif) or siRNA for LTCC in the presence or absence of DOX (10 μM) for 24 hours. (**c**) The resting levels of intracellular Ca^2+^ were expressed as percentages by assigning the levels of intracellular Ca^2+^ obtained with ionomycin in the presence and absence of extracellular Ca^2+^, to be 100% and 0%, respectively. Cap-tipped lines indicate intracellular Ca^2+^ levels (n = 5–8). (**d**) Representative immunoblots of CaMKII, phosphorylated CaMKII, and GAPDH in NRVMs treated with or without BAPTA (2 μM) in the presence or absence of DOX (10 μM) for 24 hour (n = 5). (**e**) Quantitative analysis of phosphorylated CaMKII in each group (n = 5). The experiment was conducted 2 times. **P < 0.01: post-hoc Tukey’s comparison test.

To investigate whether intracellular Ca^2+^ levels are associated with cardiomyocyte death in DOX-induced cardiotoxicity, we measured resting intracellular Ca^2+^ concentration by Fura-2 fluorometry (Fig. 4a). DOX treatment increased resting intracellular Ca^2+^ concentration in NRVMs, which was significantly suppressed by either nifedipine or amlodipine (Fig. 4b,c Supplementary Fig. 3g). Downregulation of LTCC by transduction of siRNA also attenuated DOX-induced elevation of resting intracellular Ca^2+^ concentration at the same level as nifedipine (Fig. 4b,c). Furthermore, we investigated whether intracellular Ca^2+^ levels was intimately involved in CaMKII activation. 1,2-Bis (2-aminophenoxy) ethane-N,N,N’,N’-tetraacetic acid (BAPTA, 2 μM, 24 h), an intracellular Ca^2+^ chelator, significantly suppressed DOX-induced phosphorylation of CaMKII (Fig. 4d,e). These data indicate that LTCC-related intracellular Ca^2+^ accumulation induces CaMKII activation in NRVMs treated with DOX.

### CaMKII positively regulated DOX-induced cardiomyocyte apoptosis by activating NF-κB

**Figure 5:**
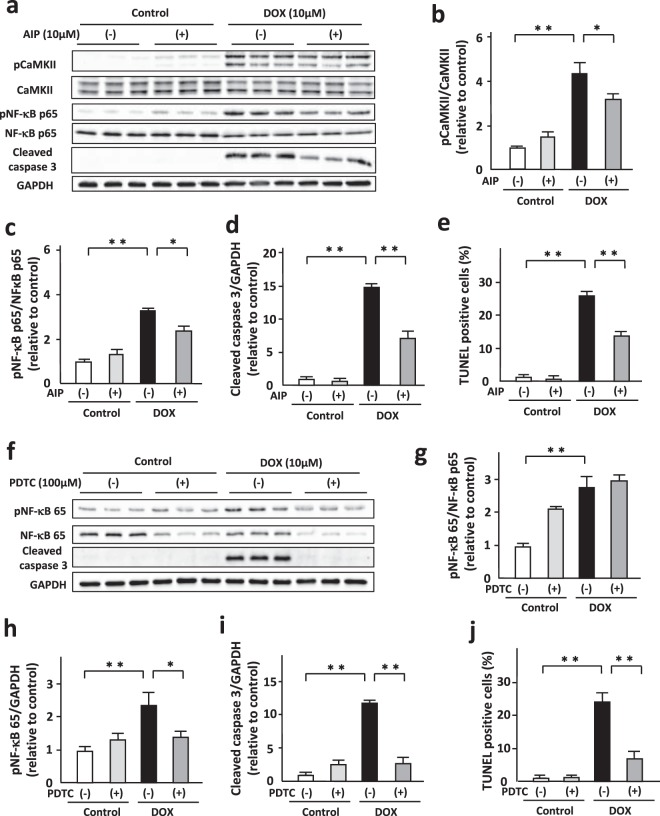
CaMKII positively regulated DOX-induced cardiomyocyte apoptosis by activating NF-κB. (**a**) Representative immunoblots of CaMKII, phosphorylated CaMKII, NF-κB, phosphorylated NF-κB, cleaved caspase 3, and GAPDH in NRVMs treated with or without AIP (10 μM, 24 h) in the presence or absence of DOX (10 μM) for 24 hours (n = 5–6). (**b**–**d**) Quantitative analysis of phosphorylated CaMKII, phosphorylated NF-κB, and cleaved caspase 3 in each group (n = 5–6). The experiment was conducted 3 times. (**e**) Apoptosis evaluated by TUNEL staining in each group (n = 5). The experiment was conducted 2 times. (**f**) Representative immunoblots of NF-κB, phosphorylated NF-κB, cleaved caspase 3, and GAPDH in NRVMs treated with or without PDTC (100 μM, 24 h) in the presence or absence of DOX (10 μM) for 24 hours (n = 5–6). (**g**–**i**) Quantitative analysis of phosphorylated NF-κB and cleaved caspase 3 in NRVMs treated with or without PDTC (100 μM, 24 h) in the presence or absence of DOX (10 μM) for 24 hours (n = 5–6). The experiment was conducted 2 times. (**j**) Apoptosis evaluated by TUNEL staining in each group (n = 5). The experiment was conducted 2 times. *P < 0.05, **P < 0.01: post-hoc Tukey’s comparison test.

To elucidate molecular mechanisms of DOX-induced cardiomyocyte apoptosis, we investigated the involvement of CaMKII in regulation of NF-κB in DOX-treated cardiomyocytes. Autocamtide 2-related inhibitory peptide (AIP, 10 μM, 24 h), a selective inhibitor of CaMKII, significantly decreased phosphorylation of CaMKII in DOX-treated NRVMs (Fig. 5a,b). Importantly, AIP attenuated DOX-induced phosphorylation of NF-κB p65 without affecting its protein levels (Fig. 5a,c). Consistent with phosphorylation of NF-κB p65, DOX induced NF-κB translocation to nucleus and AIP significantly suppressed it (Supplementary Fig. 5a,b). In addition, AIP decreased cleaved caspase-3 (Fig. 5a,d) and the number of TUNEL positive cardiomyocytes after DOX treatment (Fig. 5e). These data indicate that CaMKII is a positive regulator of NF-κB activity and cardiomyocyte apoptosis. There are several CaMKII isoforms including CaMKIIα, CaMKIIβ, CaMKIIδ, and CaMKIIδ. Among them, CaMKIIδ is a major isoform in the heart. We investigated the role of CaMKIIδ in NF-kB phosphorylation by using small interfering RNA. Knockdown of CaMKIIδ decreased phosphorylation of NF-κB (Supplementary Fig. 6a–d).

Next, we investigated whether CaMKII regulates other factors known to be related to apoptosis. DOX induced phosphorylation of extracellular signal-regulated kinase (ERK), p38 mitogen-activated protein kinase (MAPK), and c-jun N-terminal kinase (JNK) and increased p53 protein levels, but AIP did not change them (Supplementary Fig. 7a–e). Furthermore, we evaluated signaling molecules involved in mitochondrial function, autophagy, and endoplasmic reticulum (ER) stress. Although DOX decreased mitochondrial transcription factor A (TFAM), PTEN-induced kinase 1 (PINK1), and C/EBP homologous protein (CHOP) and increased phosphorylation of Drp1, AIP did not affect these changes (Supplementary Fig. 7f–i,l). Both DOX and AIP did not affect Parkin and LC3-II (Supplementary Fig. 7f,j,k). These data suggest that NF-κB is a selective target of CaMKII in DOX-treated cardiomyocytes.

Ammonium pyrrolidinedithiocarbamate (PDTC, 100 μM, 24 h), a NF-κB inhibitor, significantly inhibited DOX-induced phosphorylation of NF-κB (Fig. 5f,g). It attenuated DOX-induced increases in cleaved caspase 3 (Fig. 5f,h) and the number of TUNEL-positive NRVMs (Fig. 5i). Taken together, CaMKII mediates DOX-induced cardiomyocyte apoptosis by activating NF-κB.

### Blockade of LTCC suppressed CaMKII-NF-κB pathway in DOX-treated hearts *in vivo*

Next, we investigated the role of LTCC in DOX-treated hearts. Mice were treated with PBS or DOX (18 mg/kg via 3 intravenous injections over 1 week) with or without a subpressor dose of nifedipine (10 mg/kg/day) (Supplementary Fig. 8). DOX and nifedipine did not change body weight at 7 and 14 days after DOX injection (Supplementary Fig. 9a). They did not alter food consumption (Supplementary Fig. 9b) during 14 days and systolic blood pressure during at 14 days (Supplementary Fig. 9c). In addition, DOX did not cause death.

**Figure 6:**
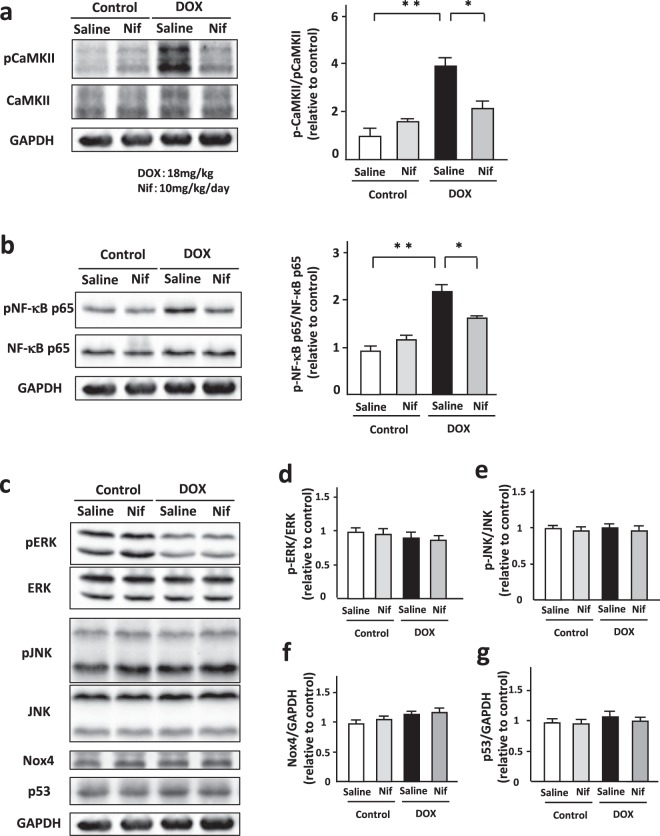
Blockade of LTCC suppressed CaMKII-NF-kB pathway in DOX-treated hearts. (**a**) Representative immunoblots and quantitative analysis of CaMKII, phosphorylated CaMKII, and GAPDH in DOX (3 doses of DOX at 6 mg/kg body weight every third day for 1 week) or control vehicle (phosphate-buffered saline: PBS) treated-C57B/6 J mouse hearts subjected to either nifedipine (Nif, 10 mg/day/day) or saline for 9 days (n = 5). (**b**) Representative immunoblots and quantitative analysis of NF-κB, phosphorylated NF-κB, cleaved caspase 3, and GAPDH in each group (n = 5). The experiment was conducted 3 times. (**c**–**g**) Representative immunoblotsa and quantitative analysis of ERK, phosphorylated ERK, JNK, phosphorylated JNK, Nox4, p53, and GAPDH in each group (n = 5).

Nine days after DOX injection, DOX induced phosphorylation of CaMKII and NF-κB p65 in the heart and nifedipine suppressed both of them (Fig. 6a,b). We evaluated other cell death related factors such as ERK, JNK, Nox4, and p53 in the heart. DOX tended to decrease phosphorylation of ERK, but nifedipine did not affect them (Fig. 6c,d). The expression levels of JNK, p53, and Nox4 and phosphorylation of JNK were not altered by DOX and nifedipine in the heart (Fig. 6c,e,f,g). These data indicate that blockade of LTCC suppressed CaMKII-NF-κB pathway in DOX-treated hearts.

### Blockade of LTCC ameliorated DOX-induced myocardial injury and apoptosis in mice

**Figure 7:**
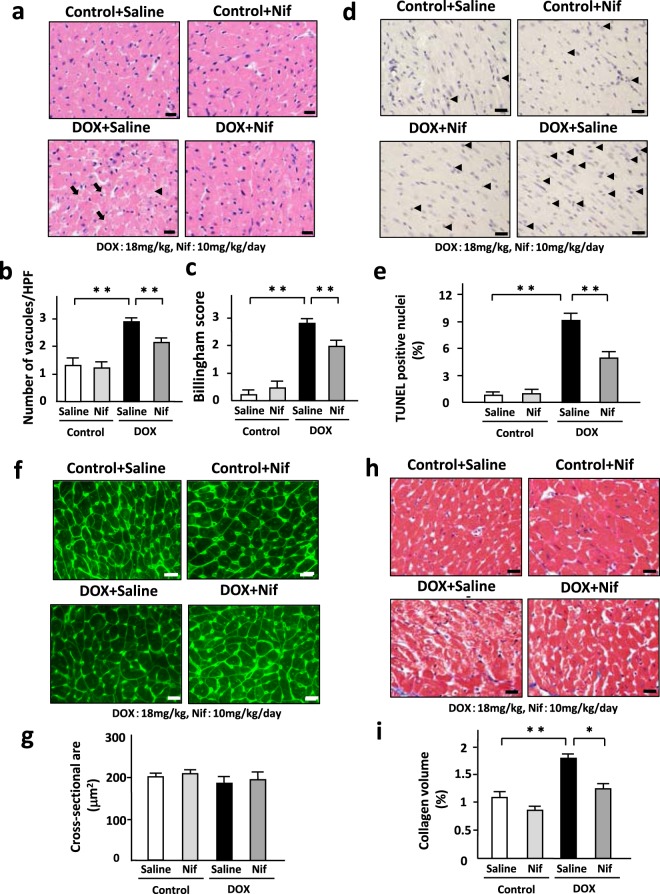
Blockade of LTCC ameliorated DOX-induced myocardial injury and apoptosis in mice. (**a**) HE stained section in DOX (3 doses of DOX at 6 mg/kg body weight every third day for 1 week) or control vehicle (phosphate-buffered saline: PBS) treated-C57B/6 J mouse hearts subjected to either nifedipine (Nif, 10 mg/day/day) or saline for 14 days. Arrows indicate cytoplasmic vacuolization and arrowheads indicate myofibrillar loss. (**b**) Cardiomyocyte injury as assessed by number of cytoplasmic vaculolization (n = 6). (**c**) Cardiomyocyte death as assessed by Billingham score in the heart (n = 5). (**d**) TUNEL stained heart section in each group. Arrow heads indicate TUNEL-positive nuclei. (**e**) The percentage of TUNEL-positive nuclei in the heart (n = 5). (**f**) WGA-stained heart section in each group. (**g**) Cardiomyocyte hypertrophy as assessed by cross-sectional area in each group (n = 5). (**h**) Masson-trichrome-stained heart section in each group. (**i**) Interstitial fibrosis as assessed by collagen volume in each group (n = 5). *P < 0.05, **P < 0.01: post-hoc Tukey’s comparison test.

We assessed DOX-induced cardiomyocyte injury by number of cytoplasmic vaculolization and cardiomyocyte death by Billingham score. Nifedipine significantly prevented DOX-induced cardiomyocyte injury (Fig. 7a,b) and cardiomyocyte death (Fig. 7a,c). It suppressed DOX-induced apoptosis as evaluated by TUNEL staining in the heart (Fig. 7d,e). Cardiomyocyte cross-sectional area was not altered by DOX or nifedipine (Fig. 7f,g). Collagen volume fraction was increased in DOX-treated hearts, which was significantly attenuated by nifedipine (Fig. 7h,i).

### Blockade of LTCC attenuated DOX-induced cardiac dysfunction and loss of heart weight in mice

**Figure 8:**
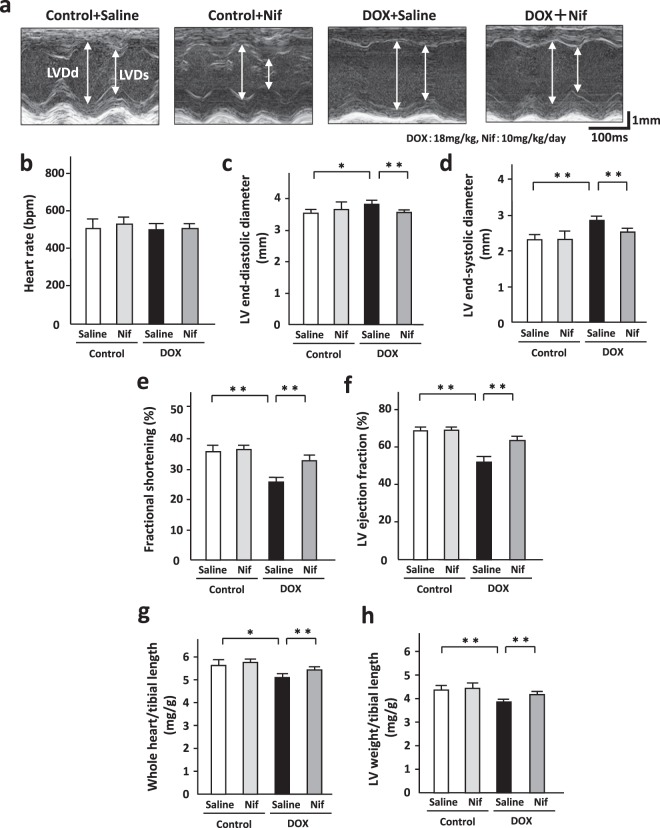
Blockade of LTCC attenuated DOX-induced cardiac dysfunction and loss of heart weight in mice. (**a**) The representative echocardiographic images of DOX (3 doses of DOX at 6 mg/kg body weight every third day for 1 week) or control vehicle (phosphate-buffered saline: PBS) treated-C57B/6 J mouse hearts subjected to either nifedipine (Nif, 10 mg/day/day) or saline for 14 days. Long two-way arrows and short two-way arrows indicate left ventricular end-diastolic diameter (LVDd) and left ventricular end-systolic diameter (LVDs), respectively. (**b**–**f**) Heart rate, LVDd and LVDs, fractional shortening, and LV ejection fraction measured by echocardiography at day14 in each group (n = 6–14). (**g**,**h**) Heart weight to tibial length (TL) ratio and LV weight to TL ratio in each group (n = 6–14). *P < 0.05, **P < 0.01: post-hoc Tukey’s comparison test.

We evaluated cardiac function by echocardiography (Fig. 8a). Heart rate was comparable in groups (Fig. 8b). DOX-treated mice had greater left ventricular (LV) end-diastolic diameter (Fig. 8c) and end-systolic diameter (Fig. 8d) and lower fractional shortening (Fig. 8e) and LV ejection fraction (Fig. 8f) than control mice. Nifedipine attenuated these DOX-induced LV dilatation (Fig. 8c,d) and dysfunction (Fig. 8e,f). LV wall thicknesses were comparable in groups (Control + Saline: 0.62 ± 0.02 mm, Control + Nif: 0.57 ± 0.02 mm, DOX + Saline: 0.56 ± 0.01 mm, DOX + Nif: 0.63 ± 0.02 mm). DOX-treated mice showed lower whole heart weight and LV weight and nifedipine significantly ameliorated these decreases (Fig. 8g,h).

## Discussion

The present study provided three novel findings. First, LTCC was a key regulator of intracellular Ca^2+^ and activation of CaMKII and NF-κB in DOX-induced cardiomyocyte apoptosis. Second, CaMKII positively regulated NF-κB activity in DOX-induced cardiomyocyte apoptosis. Finally the blockade of LTCC attenuated DOX-induced cardiomyopathy by suppressing CaMKII-NF-κB pathway (Supplementary Fig. 10). To our knowledge, the present study is the first report demonstrating the role of LTCC in DOX-induced cardiomyopathy and its downstream signaling.

CaMKII, a serine/threonine-specific protein kinase, plays a central role in development of cardiac hypertrophy and failure^12^. A recent study has shown that activation of CaMKII mediates DOX-induced cardiomyocyte death including apoptosis and cardiomyopathy^13^. CaMKII is activated by an increase in intracellular Ca^2+^ concentration^14^. There are several sources of intracellular Ca^2+^ such as LTCCs coupled with Ca^2+^-induced Ca^2+^ release from the ryanodine receptor, T-type Ca^2+^ channel, transient receptor potential channel, and IP_3_ receptor channel^27^. Among them, LTCCs are the primary source of Ca^2+^ influx to initiate cardiac excitation-contraction coupling^28,29^. Ca^2+^ influx from LTCCs mediates intracellular signaling that underlie cardiac hypertrophy^30,31^. In addition, previous studies have shown that DOX increased Ca^2+^ concentration in cardiomyocytes^10,11^. In the present study, both pharmacological blockade and gene knockdown of LTCC suppressed DOX-induced intracellular Ca^2+^ elevation and CaMKII activation in cardiomyocytes. Although DOX did not affect the expression levels of LTCC, it significantly increased phosphorylation of LTCC (Supplementary Fig. 2). These data suggest that intracellular Ca^2+^ levels regulated by LTCC is implicated in CaMKII activity in DOX-induced cardiotoxicity. The pathological role of LTCC in cardiovascular diseases, especially in cardiac hypertrophy, has been reported to be controversial^32,33^. Our results demonstrated a critical role of LTCC in CaMKII activity and a protective effect of its blockade against DOX-induced cardiomyopathy. Among 4 isoforms of CaMKII (α, β, δ, and γ subunit), CaMKIIδ is predominantly expressed in the heart^34^. Knockdown of CaMKIIδ suppressed DOX-induced phosphorylation of NF-κB (Supplementary Fig. 6), indicating that CaMKIIδ is a critical isoform mediating DOX-induced cardiotoxicity. However, further investigations are needed to determine the distinct role of other CaMKII isoforms in DOX-induced cardiotoxicity.

NF-κB is a multifunctional transcriptional factor, which is intimately involved in cardiomyocyte death^20,35^ and cardiac remodeling^36^. The present study demonstrated that DOX evoked NF-κB activity, which was suppressed by blockade of LTCC (Figs 2a,c, 6b). Inhibition of NF-κB attenuated DOX-induced increases in cleaved caspase 3 and cardiomyocyte apoptosis (Fig. 5I,j). Thus, the blockade of LTCC ameliorated DOX-induced cardiotoxicity probably due to the suppression of not only CaMKII but also NF-κB. CaMKII is known to have several downstream targets such as histone deacetylase 4 (HDAC4) and nuclear factor of activated T cells (NFAT) in cardiac hypertrophy^37^, however, its downstream targets in DOX-induced cardiomyopathy have not been revealed yet. We here found that the inhibition of CaMKII activity by AIP, a selective CaMKII inhibitor, suppressed DOX-induced NF-κB activation (Fig. 5a,c). In addition, knockdown of CaMKIIδ suppressed DOX-induced NF-κB activation (Supplementary Fig. 6). These findings suggest that CaMKII, especially CaMKIIδ, is a positive regulator of NF-κB and CaMKII-NF-κB axis critically mediates cardiomyocyte apoptosis in DOX-induced cardiotoxicity. There are several reports regarding association between NF-kB and apoptosis. Li *et al*. demonstrated that NF-κB activation induced apoptosis through upregulation of PUMA^38^. In addition, it has been reported that NF-kB induces apoptosis via inflammation^21^. However, further investigation is required to determine precise mechanisms regulating phosphorylation of NF-kB by CaMKII.

Mitochondrial function^26^, autophagy^25^, and ER stress^39^ are known to be implicated in DOX-induced cardiotoxicity. However, AIP did not affect DOX-induced changes of TFAM, PINK1, CHOP and phosphorylation of Drp1 (Supplementary Fig. 7g–i,l). Although phosphorylation of JNK^40^, ERKs/p53 signal^41^, and Nox4^42^ was reported to be involved in DOX-induced cardiomyocyte apoptosis, our results demonstrated that AIP or nifedipine did not affect DOX-induced changes of these proteins (Fig. 6c-g and Supplementary Fig. 7a–e). Thus, blockade of LTCC might attenuate DOX-induced cardiomyopathy by selectively suppressing CaMKII-NF-κB axis.

Interestingly, nifedipine attenuated not only apoptosis (Fig. 7d,e) but also interstitial fibrosis (Fig. 7h,i) in DOX-treated hearts. Nifedipine may primarily prevent apoptosis and secondarily attenuate reactive fibrosis in DOX-induced cardiomyopathy. In addition, it might directly act on non-cardiomyocytes such as fibroblasts.

A low-dose DOX treatment did not affect body weight and food consumption. The heart treated with a low-dose DOX demonstrated LV dilatation, impaired LV dysfunction, loss of LV weight, and increases in cardiomyocyte injury, apoptosis, and interstitial fibrosis (Fig. 7a–e,h,i). These phenotypes are observed in human DOX-induced cardiomyopathy, indicating that a low-dose DOX model is equivalent to this disease in human. The beneficial effects of nifedipine shown in this study were not due to its blood pressure lowering effect or nutrition preserving effect because it did not alter blood pressure, body weight, and food consumption.

In this study, we used nifedipine and amlodipine, which are most widely prescribed LTCC blockers for treatment of hypertension. Pleiotropic effects of these drugs can not be completely excluded. However, nifedipine and amlodipine similarly suppressed DOX-induced elevation of intracellular Ca^2+^ concentration and activation of CaMKII and NF-κB in cardiomyocytes. Furthermore, gene knockdown of LTCC also significantly suppressed them. These findings based on different types of intervention in LTCC indicate that CaMKII and NF-κB are downstream targets of LTCC-related intracellular Ca^2+^ in DOX-induced cardiomyopathy.

In general, short-acting Ca^2+^ channel blockers are not recommended for LV dysfunction and heart failure because of their hypotensive potential. Long-acting Ca^2+^ channel blocker might be better for DOX-induced cardiomyopathy with LV dysfunction. Moreover, the continuous way of administration may overcome the hypotension issue.

In conclusions, blockade of LTCC attenuates DOX-induced cardiomyocyte apoptosis by suppressing intracellular Ca^2+^ abnormalities and CaMKII-NF-κB pathway. LTCC blocker protects the heart against DOX-induced cardiotoxicity *in vivo* by suppressing CaMKII-NF-κB pathway. Therapeutic strategy designed to interfere with this pathway by LTCC blocker might be beneficial in DOX-induced cardiomyopathy.

## Methods

### Reagents

Doxorubicin (D1515), nifedipine (N7634), autocamtide 2-related inhibitory peptide (AIP, A4308), and ammonium pyrrolidinedithiocarbamate (PTCD, P8765) were purchased from sigma. Amlodipine (A2353) was purchased from Tokyo Chemical Industry. 1,2-Bis (2-aminophenoxy) ethane-N,N,N’,N’-tetraacetic acid (BAPTA, ab120503) was obtained from abcam.

### Primary culture of neonatal rat ventricular myocytes

Primary cultures of ventricular cardiac myocytes were prepared from 1-day-old Sprague-Dawley rats (Kyudo Inc, Japan). A cardiac myocyte-rich fraction was obtained by centrifugation through a discontinuous Percoll gradient as described previously^43^.

### siRNA and transfection

Silencing of L-type Ca^2+^ channel (LTCC) and CaMKIIδ gene expressions in primary neonatal rat cardiomyocytes were achieved by the small interfering RNA (siRNA) technique. The sequences of the siRNA duplexes were selected from the coding regions of the target mRNAs. Silencer select siRNA specific to decrease the expression of rat LTCC and CaMKIIδ mRNA were purchased from Themo Fisher Scientific. The sense strand of siRNA used to silence the rat LTCC gene (LTCC siRNA) and CaMKIIδ gene (CaMKIIδ siRNA) were CCUGCGAUAUGACAAUAGA, and GCAACUUAGUGGAAGGGAUTT, respectively. Transfection of cultured cardiomyocytes was carried out by Lipofectamine RNAiMAX (Themo Fisher Scientific) according to proposed protocol.

### Measurement of resting intracellular calcium concentration by Fura-2 fluorometry

Cardiomyocytes plated on 35-mm dishes were loaded with fura-2 by incubation in DMEM containing 5 μM fura-2 acetoxymethylester (Dojindo Laboratories, Kumamoto, Japan) and 1 mM at 37 °C in a chamber containing 5% CO_2_ for 15 minutes, as described previously^44,45^. Probenecid was used to prevent extracellular leakage of fura-2 by inhibiting anion transporter. After fura-2 loading, the cells were equilibrated in HEPES-buffered saline (HBS; 10 mM HEPES, pH 7.4, 135 mM NaCl, 5 mM KCl, 1 mM, CaCl_2_, 1 mM MgCl_2_, and 5.5 mM D-glucose) at room temperature for 10 min before initiating the fluorometry. Changes in the fura-2 fluorescence ratio (excitation at 340 and 380 nm; emission at 500 nm) in the cell population were monitored as an indication of intracellular Ca^2+^ concentration using a frontsurface fluorometer (CAM230-OF2), as described previously^44,45^. The resting levels of intracellular Ca^2+^ levels were expressed as percentages by assigning the levels of intracellular Ca^2+^ obtained with ionomycin, (a calcium ionophore, LKT Laboratories, St. Paul, MN) in the presence and absence of intracellular and extracellular Ca^2+^, to be 100% and 0%, respectively. EGTA was used to chelate intracellular calcium. In addition, probenecid was used to prevent extracellular leakage of fura-2 by inhibiting anion transporter.

### Immunoblot analyses

Cardiomyocyte lysates and heart homogenates were prepared in RIPA lysis buffer containing Tris-HCl, NaCl, NP-40, sodium deoxycholae, and SDS. For immunoblot analyses, we used monoclonal antibodies against CaMKII (#4436, Cell Signaling Technology), NF-kB (#8242, Cell Signaling Technology), phospho-CaMKII (Thr286) (#12716, Cell Signaling Technology), phospho-NF-κB (Ser536) (#3033, Cell Signaling Technology), cleaved caspase 3 (#9664, Cell Signaling Technology), phospho-JNK (Thr183/Tyr185) (#4668, Cell Signaling Technology), p53 (#32532, Cell Signaling Technology), CHOP (#5554, Cell Signaling Technology), PINK1 (ab75487, abcam), DRP1 (611112, BD Bioscience), phosphor-DRP1 (#4494), and GAPDH (sc32233, Santa Cruz Biotechnology), and polyclonal antibodies against ERK (#9102, Cell Signaling Technology), phospho-ERK (Thr202/Tyr204) (#9101, Cell Signaling Technology), JNK (#9252, Cell Signaling Technology), Nox4 (NB11058849, Novus), Parkin (ab15954, abcam), LTCC (#ACC-003, alomone labs), phospho LTCC (A010–70, Badrilla), and mtTFAM (sc23588, Santa Cruz Biotechnology).

### Immunostaining

Cardiomyocytes grown glass plate dish were washed 3 times with PBS. The cells were fixed with 4% paraformaldehyde and washed 3 times with PBS. The cells were then treated with 0.1% Triton X-100 for 15 minutes and washed 3 times with PBS. Cells were blocked with PBS containing 1% bovine serum albumin for 60 minutes and stained with antibodies as indicated.

### TUNEL staining

TUNEL staining was conducted as described^46^. Deparaffinized sections were incubated with proteinase K and DNA fragments were labeled with fluorescein-conjugated dTUP using *in situ* Apoptosis Detection kit (MK500, Takara). Nuclear density was determined by manual counting of DAPI-stained nuclei in 10 fields for each animal using a 40x objective.

### LDH release assay

Lactate dehydrogenase (LDH) release into the media from damaged cells were measured by LDH cytotoxicity assay kit (299–50601, wako).

### Doxorubicin-induced cardiomyopathy model

To create a mouse model mimicking human doxorubicin cardiomyopathy, 9 to 10-weeks-old C57BL/6 J mice were treated with 3 doses of DOX at 6 mg/kg body weight intravenously via tail vein injections every third day for 1 week as described^26^. Continuous infusion of nifedipine (10 mg/kg/day) or control vehicle delivery was conducted using a miniosmotic pump (model 2002, Alzet). Nifedipine was prepared at a concentration calculated to deliver an average of 10 mg/kg/day during a 14-day infusion period. Control mice received pumps filled with vehicle (dimethyl sulfoxide) alone. Immunoblot analysis was conducted by using hearts at 9 days after starting saline or DOX injection. After 14 days, echocardiography was performed and then the heart was extracted for histological analysis (Supplementary Fig. 4). All procedures involving animals and animal care protocols were approved by the Committee on Ethics of Animal Experiments of the Kyushu University Graduate School of Medicine and Pharmaceutical Sciences (A29–390), and were performed in accordance with the Guideline for Animal Experiments of Kyushu University and the Guideline for the Care and Use of Laboratory Animals published by the US National Institutes of Health (revised in 2011).

### Echocardiography

Under light anesthesia with 1–2% isoflurane, two-dimensional targeted M-mode images were obtained from the short axis view at the papillary muscle level using a Vevo 2100 ultrasonography system (Visual Sonics, Toronto, Canada) as previously described^47^. Fractional shortening is calculated from: %FS = [(diastolic LV diameter—systolic LV diameter)/diastolic LV diameter) × 100]. LV wall thickness was calculated as average of interventricular septum thickness and posterior wall thickness.

### Histological analyses

The LV accompanied by the septum was cut into base and apex portions, fixed with 10% formalin and submitted for hematoxylin and eosin staining. Myocardial injury was evaluated by Billingham score and cytoplasmic vaculolization as previously reported^48,49^. Briefly, Billingham scores were based on the percentage of myocytes showing cytoplasmic vacuolization and/or myofibrillar loss, and graded from 0 to 3 as follows: 0, no damaged cells; 1, <5%; 1.5, 5–15%; 2.0, 16–25%; 2.5, 26–35%; and 3, >35% damaged cells. Cytoplasmic vacuolization were assessed around 20 microscopic fields randomly selected in LVs per section, 3 sections from each mouse. Myocyte cross-sectional area was evaluated by mid-LV stained with wheat germ agglutinin (WGA) as described previously^50^. Collagen volume was determined by quantitative morphometry of tissue sections from the mid-LV stained with Masson’s trichrome as described previously^51^.

### Statistical analysis

All values are expressed as mean ± SEM. Statistical analyses were performed using ANOVA followed by a post-hoc Tukey’s comparison test. P < 0.05 was considered to be statistically significant.

## Supplementary information

Sup fig and Fig legend

## Data Availability

All data generated or analysed during this study are included in this published article (and its supplementary information files).
