# Supplementary material for: Blockade of L-type Ca2+ channel attenuates doxorubicin-induced cardiomyopathy via suppression of CaMKII-NF-κB pathway
Source: Sci Rep. 2019 Jul 8;9:9850. doi: 10.1038/s41598-019-46367-6 (PMC6614470; doi:10.1038/s41598-019-46367-6)
Supplement: Supplementary file 1 — Sup fig and Fig legend [file 41598_2019_46367_MOESM1_ESM.pdf]

**Blockade of L-type $Ca^{2+}$ channel attenuates doxorubicin-induced cardiomyopathy via suppression of CaMKII-NF-κB pathway**

Soichiro Ikeda[1], Shouji Matsushima[2*], Kosuke Okabe[1], Masataka Ikeda[1], Akihito Ishikita[1], Tomonori Tadokoro[1], Nobuyuki Enzan[1], Taishi Yamamoto[1], Masashi Sada[1], Hiroko Deguchi[1], Sachio Morimoto[3], Tomomi Ide[4], and Hiroyuki Tsutsui[1]

[1]Department of Cardiovascular Medicine, Faculty of Medical Sciences, Kyushu University, Fukuoka, Japan

[2]Department of Cardiovascular Medicine, Kyushu University Hospital, Fukuoka, Japan

[3]Department of Health Sciences Fukuoka, International University of Health and Welfare, Okawa, Japan

[4]Department of Experimental and Clinical Cardiovascular Medicine, Graduate School of Medical Sciences, Kyushu University, Japan

[*]Address correspondence to:

Shouji Matsushima, M.D., Ph.D.

Department of Cardiovascular Medicine,

Kyushu University Hospital,

3-1-1 Maidashi Higashi-ku Fukuoka 812-8582, Japan

Phone: +81-92-642-5357, FAX: +81-92-642-5374

e-mail: shouji-m@cardiol.med.kyushu-u.ac.jp

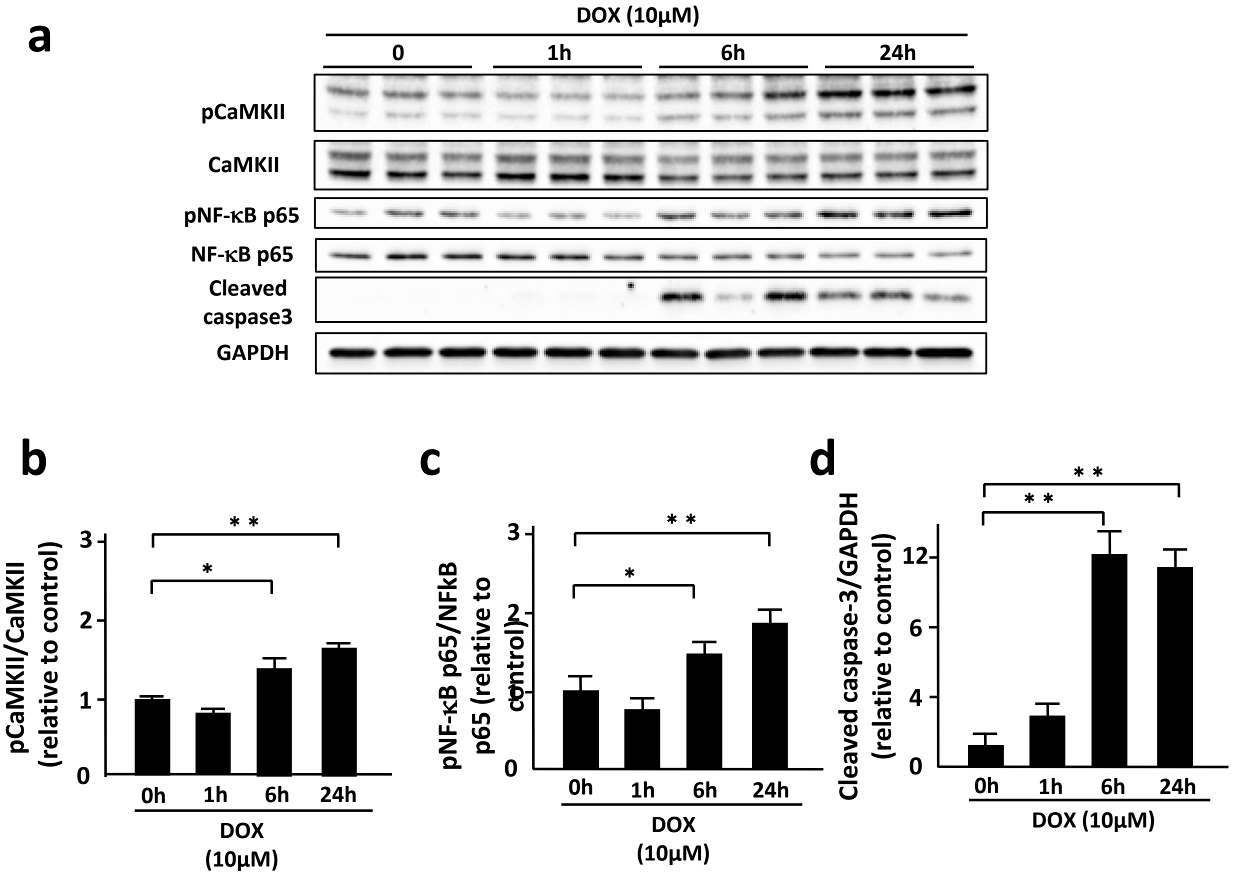


**Supplementary Figure 1. DOX induced phosphorylation of CaMKII and NF-κB and increased cleaved caspase 3 in cardiomyocytes in a time-dependent manner. (a)** Representative immunoblots of CaMKII, phosphorylated CaMKII, NF-kB, phosphorylated NF-kB, cleaved caspase 3, and GAPDH in NRVMs in indicated time point (n=5). **(b-d)** Quantitative analysis of phosphorylated CaMKII, phosphorylated NF-kB, and cleaved caspase 3 in NRVM in indicated time point (n=5). The experiment was conducted 2 times. *P<0.05: post-hoc Tukey's comparison test.

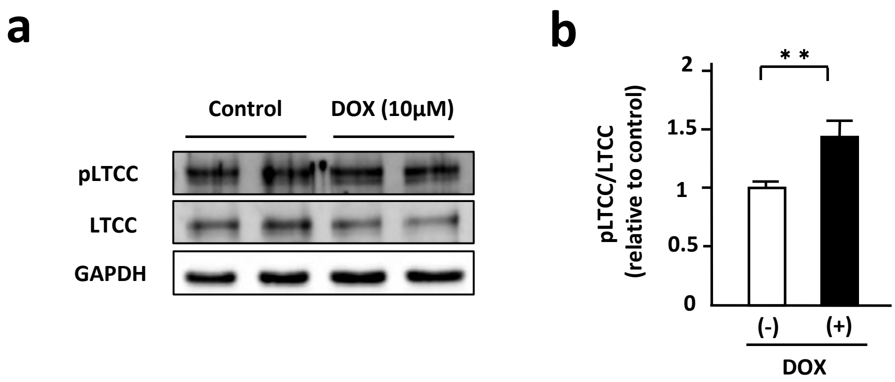


**Supplementary Figure 2. DOX increased phosphorylation of LTCC. (a)** Representative immunoblots of LTCC, phosphorylated LTCC, and GAPDH in NRVMs in indicated groups (n=5). **(b)** Quantitative analysis of LTCC, phosphorylated LTCC, and GAPDH in NRVM in indicated groups (n=5). The experiment was conducted 2 times. *P<0.05: post-hoc Tukey's comparison test.

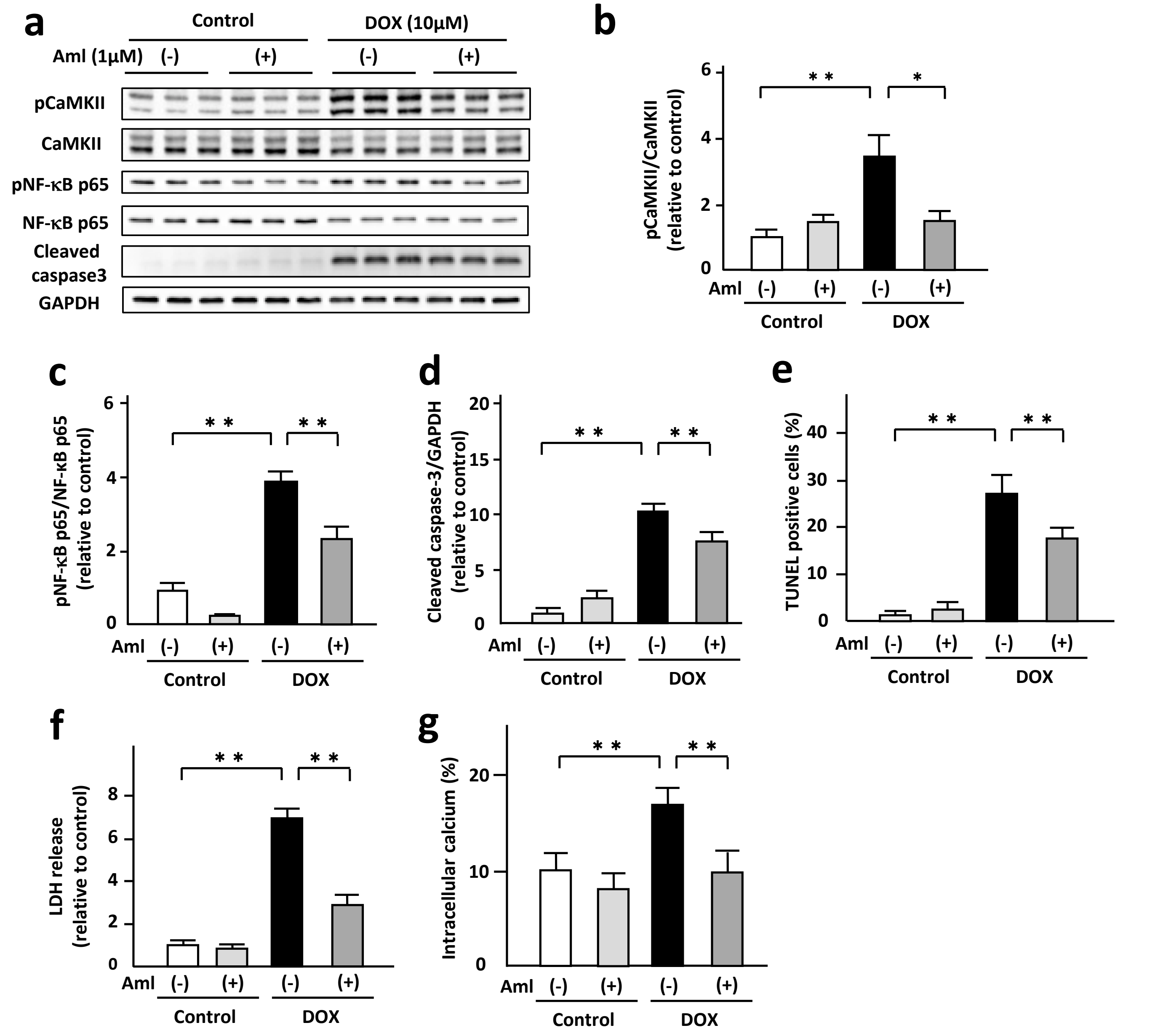


**Supplementary Figure 3. Amlodipine suppressed DOX-induced phosphorylation of CaMKII and NF-κB, increases in cleaved caspase 3, and cardiomyocyte apoptosis. (a)** Representative immunoblots of CaMKII, phosphorylated CaMKII, NF-kB, phosphorylated NF-kB, cleaved caspase 3, and GAPDH in NRVMs treated with or without amlodipine (Aml, 1mM) in the presence or absence of DOX (10mM) for 24 hours (n=5-6). **(b-d)** Quantitative analysis of phosphorylated CaMKII, phosphorylated NF-kB, and cleaved caspase 3 in each group (n=5-6). The experiment was conducted 2 times. **(e)** Apoptosis evaluated by TUNEL staining in each group (n=5-6). The experiment was conducted 2 times. **(f)** LDH release, a marker of necrosis, evaluated by LDH cytotoxicity assay in each group (n=5-6). The experiment was conducted 2 times. **(g)** The resting levels of intracellular $Ca^{2+}$ levels in each group (n=5-6).  **P<0.01: post-hoc Tukey's comparison test.

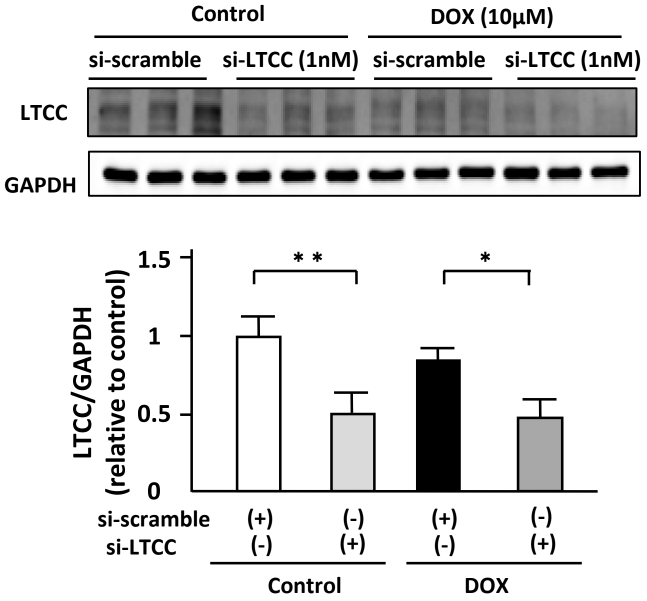


**Supplementary Figure 4. Expression levels of LTCC in cardiomyocytes.** Immunoblot analysis of Representative immunoblots and quantitative analysis of LTCC and GAPDH in NRVMs after RNA interference-mediated silencing of LTCC (n=5-6). The experiment was conducted 2 times. *P<0.05, **P<0.01: post-hoc Tukey's comparison test.

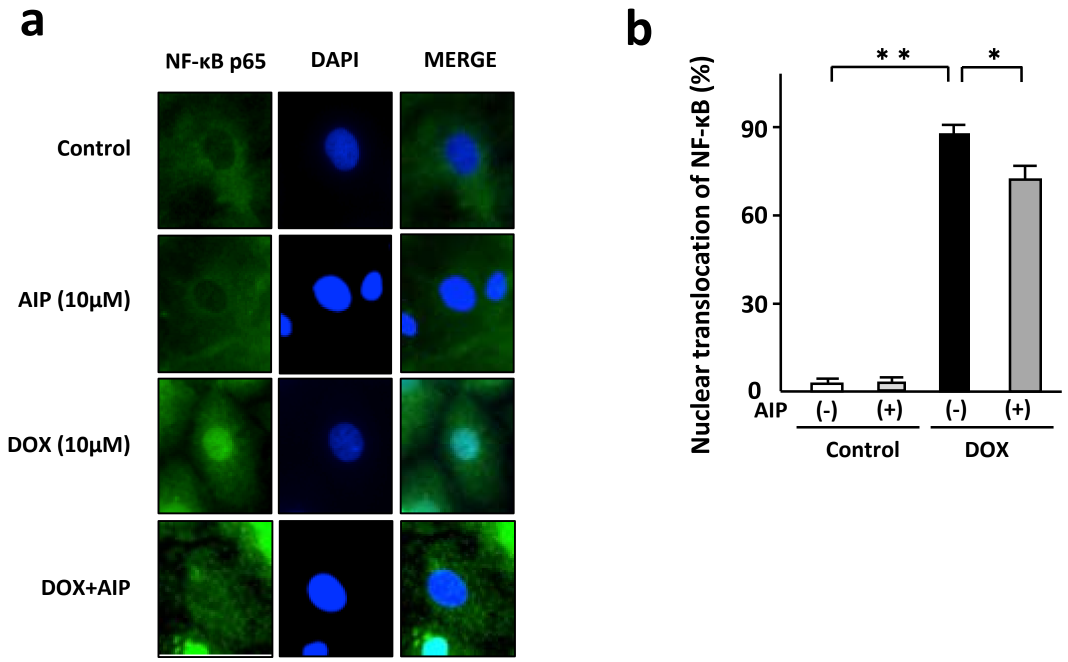


**Supplementary Figure 5. AIP prevented DOX-induced nuclear translocation of NF-κB in cardiomyocytes. (a)** Representative images of NF-kB (green) and DAPI (blue) in NRVMs in indicated groups (n=5). **(b)** Quantitative analysis of nuclear translocation of NF-kB in indicated groups (n=5). The experiment was conducted 2 times. *P<0.05: post-hoc Tukey's comparison test.

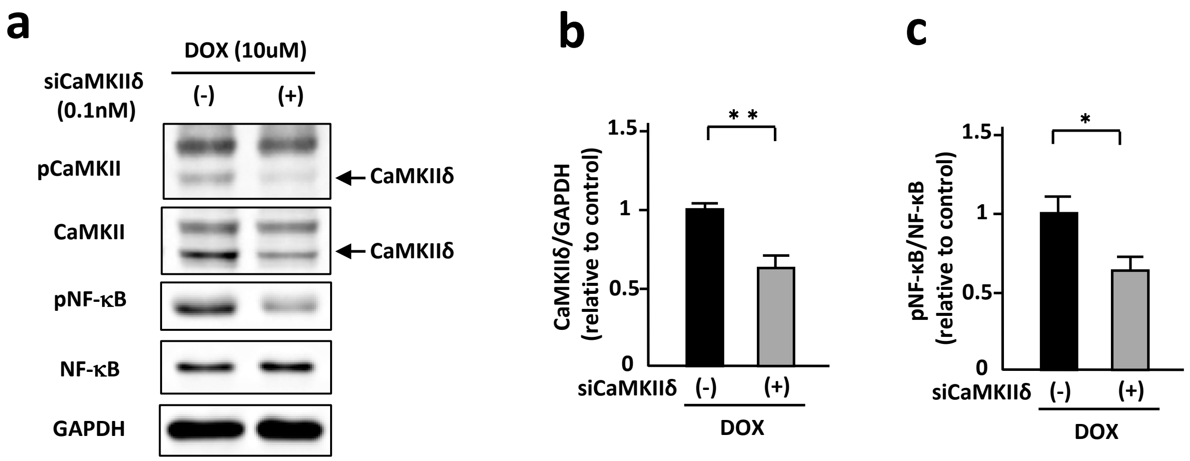


**Supplementary Figure 6. Knockdown of CaMKIIδ decreased DOX-induced phosphorylation of NF-κB. (a)** Representative immunoblots of CaMKII, phosphorylated CaMKII, NF-kB, phosphorylated NF-kB, and GAPDH in NRVMs treated with or without siCaMKIId (0.1nM, 24h) in the presence of DOX (10mM) for 24 hour (n=6). **(b and c)** Quantitative analysis of phosphorylated CaMKIId and phosphorylated NF-kB in indicated group (n=6). The experiment was conducted 2 time.

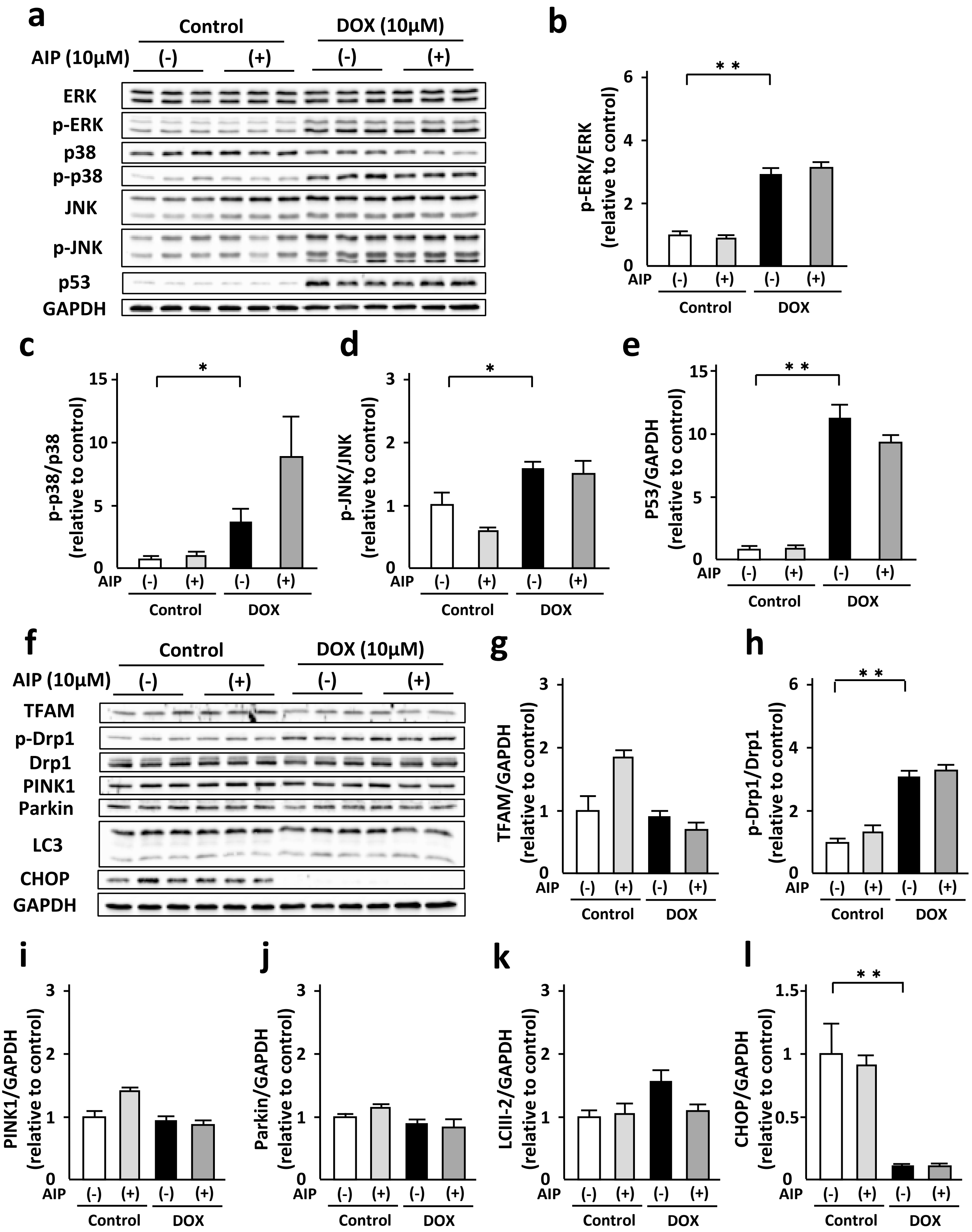
a
Control
DOX (10μM)
AIP (10μM)
(-)
(+)
ERK
p-ERK
p38
p-p38
JNK
p-JNK
p53
GAPDH
b
p-ERK/ERK
(relative to control)
AIP
Control
DOX
c
p-p38/p38
(relative to control)
d
p-JNK/JNK
(relative to control)
e
P53/GAPDH
(relative to control)
f
TFAM
p-Drp1
Drp1
PINK1
Parkin
LC3
CHOP
g
TFAM/GAPDH
(relative to control)
h
p-Drp1/Drp1
(relative to control)
i
PINK1/GAPDH
(relative to control)
j
Parkin/GAPDH
(relative to control)
k
LCIII-2/GAPDH
(relative to control)
l
CHOP/GAPDH
(relative to control)

**Supplementary Figure 7. The role of CaMKII in several signaling pathways.**
**(a-e)** Representative immunoblots and quantitative analysis of ERK, phosphorylated ERK, p38, phosphorylated p38, JNK, phosphorylated JNK, p53, and GAPDH in NRVMs treated with or without AIP (10mM, 24h) in the presence or absence of DOX (10mM) for 24 hour (n=3). **(f-l)** Representative immunoblots and quantitative analysis of TFAM, Drp1, phosphorylated Dpr1, Parkin, PINK1, LC3, CHOP, and GAPDH in NRVMs in each group (n=3). The experiment was conducted 1 time.

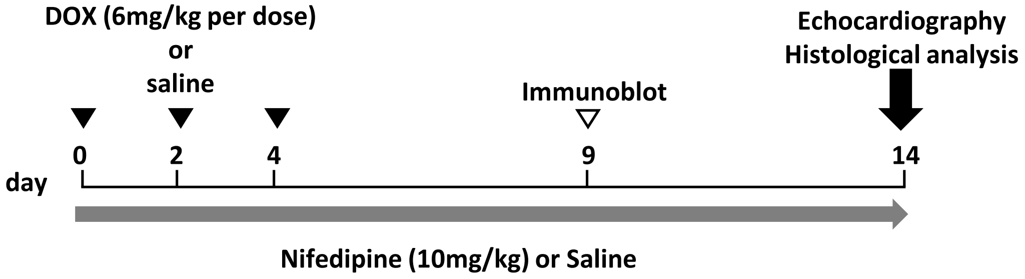


**Supplementary Figure 8. The protocol of in vivo experiment in this study.** Nine to ten-weeks-old C57BL/6J mice were treated with 3 doses of DOX at 6mg/kg body weight every third day for 1 week. Continuous infusion of nifedipine or control vehicle delivery was conducted using an osmotic minipump. Nifedipine was prepared at a concentration calculated to deliver an average of 10mg/kg/day during a 14-day infusion period. Control mice received pumps filled with vehicle (dimethyl sulfoxide) alone. Immunoblot analysis was conducted by using hearts at 9 days after starting saline or DOX injection. After 14 days, echocardiography was performed and then the heart was extracted for histological analysis.

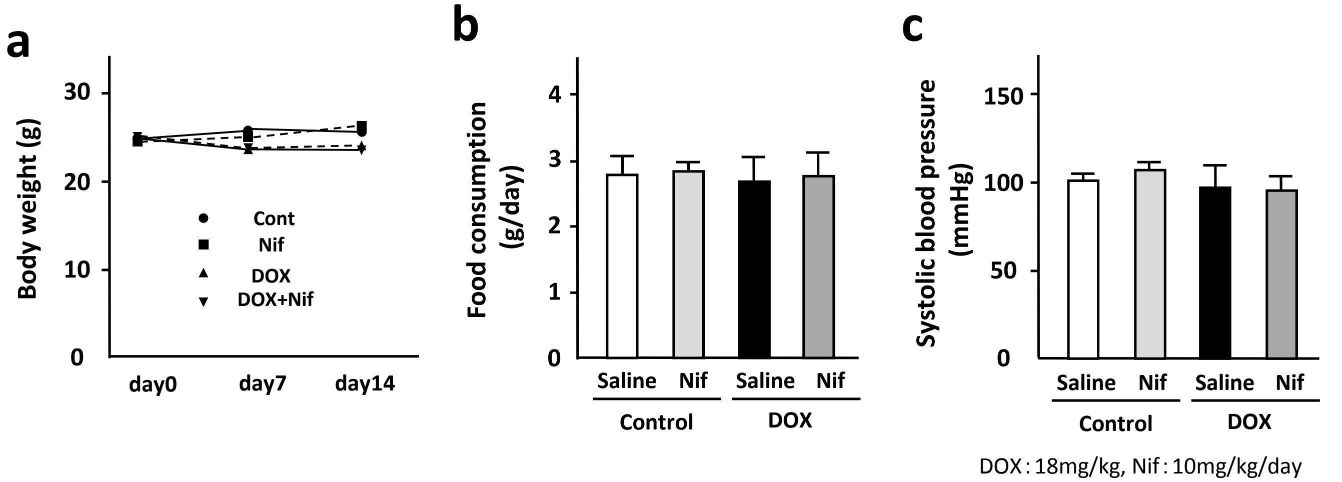


**Supplementary Figure 9. Body weight, food consumption and blood pressure in DOX-induced cardiomyopathy model. (a)** Body weight in DOX (3 doses of DOX at 6mg/kg body weight every third day for 1 week) or control vehicle (phosphate-buffered saline: PBS) treated-C57B/6J mouse hearts subjected to either nifedipine (Nif, 10mg/day/day) or saline at 7 and 14 days (n=6). **(b)** Average daily food consumption during 14 days in each group (n=6). **(c)** Systolic blood pressure measured by tail cuff at day14 in each group (n=6).

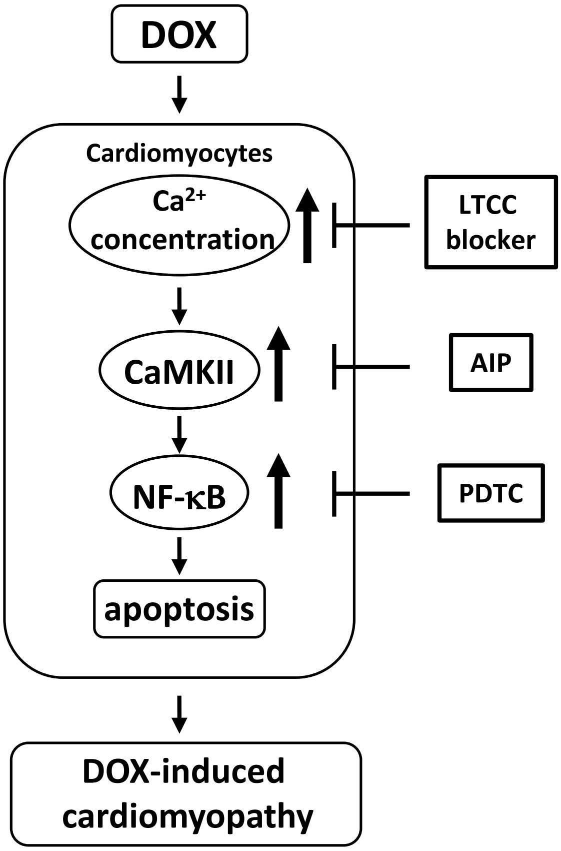


**Supplementary Figure 10. A schematic representation of signaling pathway regarding DOX-induced cardiomyopathy.** DOX induced cardiomyocytes apoptosis through CaMKII-NF-kB pathway, thereby leading to DOX-induced cardiomyopathy. LTCC-related $Ca^{2+}$ handling is intimately involved in this pathway.
